# Optimisation in X-ray and molecular imaging 2025—editorial

**DOI:** 10.1093/rpd/ncag008

**Published:** 2026-03-13

**Authors:** Anja Almén, Magnus Båth, Peter Bernhardt, Magnus Dustler, Angelica Svalkvist

**Affiliations:** Medical Radiation Physics, Department of Translational Medicine, Lund University, SE-205 02 Malmö, Sweden; Swedish Radiation Safety Authority, SE-171 16 Stockholm, Sweden; Department of Biomedical Engineering and Medical Physics, Sahlgrenska University Hospital, SE-413 45 Gothenburg, Region Västra Götaland, Sweden; Department of Medical Radiation Sciences, Institute of Clinical Sciences, Sahlgrenska Academy, University of Gothenburg, SE-413 45 Gothenburg, Sweden; Department of Biomedical Engineering and Medical Physics, Sahlgrenska University Hospital, SE-413 45 Gothenburg, Region Västra Götaland, Sweden; Department of Medical Radiation Sciences, Institute of Clinical Sciences, Sahlgrenska Academy, University of Gothenburg, SE-413 45 Gothenburg, Sweden; Medical Radiation Physics, Department of Translational Medicine, Lund University, SE-205 02 Malmö, Sweden; Department of Biomedical Engineering and Medical Physics, Sahlgrenska University Hospital, SE-413 45 Gothenburg, Region Västra Götaland, Sweden; Department of Medical Radiation Sciences, Institute of Clinical Sciences, Sahlgrenska Academy, University of Gothenburg, SE-413 45 Gothenburg, Sweden

This issue of *Radiation Protection Dosimetry* is based on contributions to the scientific conference Optimisation in X-ray and Molecular Imaging 2025 (OXMI 2025), which was held in Gothenburg, Sweden, 28–30 April 2025. The meeting was jointly organized by Sahlgrenska University Hospital, University of Gothenburg, and the Swedish Society of Radiation Physics. OXMI 2025 constituted the 6^th^ meeting in a series of conferences focusing on optimisation of medical imaging, with special emphasis on image quality evaluation and radiation safety. Previous conferences in the series have been held in Malmö, Sweden (1999, 2004, and 2009), in Gothenburg, Sweden (2015), and as an online meeting during the COVID-19 pandemic (2020).

Recent years have contributed to substantial and rapid advancements in medical imaging methodologies and hardware. Additionally, machine learning approaches have progressed substantially and today have a clinical impact on all aspects of medical imaging, e.g. image acquisition, image reconstruction, and image interpretation. Due to the constant progress in the field of medical imaging, considerable challenges concerning the development, validation, and implementation of optimisation strategies occur. These challenges constitute the primary rationale for the OXMI conference series.

The OXMI 2025 meeting gathered close to 150 participants consisting of medical physicists, radiologists, nuclear medicine physicians, engineers, radiographers and biomedical scientists, as well as representatives for authorities and manufacturers. The scientific programme consisted of both presentations based on abstracts submitted by the participants themselves and presentations held by invited experts in the fields of medical physics, radiology and nuclear medicine. In total, the programme consisted of 10 oral sessions and 1 poster session, giving a total of close to 80 scientific presentations. The presentations covered several important aspects of optimisation in medical imaging, such as, e.g. radiation protection and dosimetry in clinical practice, emerging imaging technologies, the impact of artificial intelligence and data-driven developments, algorithmic and quantitative image quality assessment, and the human perspective on image quality assessment and radiation risks.

We believe that participants of OXMI 2025, as well as readers of *Radiation Protection Dosimetry*, will welcome the opportunity to further engage with the conference contributions through these peer-reviewed proceedings.

